# Diaphragmatic thickness in chronic obstructive lung disease and relationship with clinical severity parameters

**DOI:** 10.3906/sag-1901-164

**Published:** 2019-08-08

**Authors:** Nalan OGAN, Yusuf AYDEMİR, Togay EVRİN, Gökçe Kaan ATAÇ, Ayşe BAHA, Burak KATİPOĞLU, Banu SÜZEN, Evrim Eylem AKPINAR

**Affiliations:** 1 Department of Pulmonology, Faculty of Medicine, Ufuk University, Ankara Turkey; 2 Department of Pulmonology, Faculty of Medicine, Sakarya University, Sakarya Turkey; 3 Department of Emergencies, Faculty of Medicine, Ufuk University, Ankara Turkey; 4 Department of Radiology, Faculty of Medicine, Ufuk University, Ankara Turkey; 5 Department of Pulmonology, Girne Dr. Akçiçek Hospital, Girne North Cyprus; 6 Department of Nutrition and Diet, Faculty of Medicine, Ufuk University, Ankara Turkey

**Keywords:** Chronic obstructive pulmonary disease, diaphragm, chest ultrasound

## Abstract

**Background/aim:**

Sonographic assessment of diaphragm structure and function would be a useful clinical tool in patients with chronic obstructive pulmonary disease (COPD). Our aim was to determine the muscle thickness of the diaphragm and the usefulness of clinical practice in patients with COPD.

**Materials and methods:**

The diaphragmatic thickness of 34 COPD patients and 34 healthy subjects was measured during tidal volume (Tmin) and deep inspiration (Tmax) on both sides using a B-mode ultrasound. The body mass index and the modified Medical Research Council (mMRC) index values were reported.

**Results:**

There was no correlation among TminR (P = 0.134), TminL (P = 0.647), TmaxR (P = 0.721), and TmaxL (P = 0.905) between the patients with COPD and the control group. There was also no significant difference between diaphragmatic thickness and COPD severity, respiratory function (P = 0.410), and frequency of exacerbations (P = 0.881) and mMRC (P = 0.667).

**Conclusion:**

Diaphragmatic dysfunction in COPD is related to mobility restriction rather than muscle thickness.

## 1. Introduction

Chronic obstructive pulmonary disease (COPD) is characterized by a progressive restriction of airways. Structural damage in the lung parenchyma causes pulmonary hyperinflation. This condition restricts diaphragmatic mobility in patients with severe COPD. The diaphragm flattens, reducing the upward and downward movement of the lung. In patients with severe COPD, the contraction of the auxiliary respiratory muscles, such as the sternocleidomastoid and scalene muscles, compensates for the insufficiency of diaphragm, the main respiratory muscle [1].

Loss of fat-free mass (FFM) and weakened skeletal muscles caused by factors including reduced protein production, malnutrition, increased muscular apoptosis, oxidative stress, inflammatory mediators in systemic circulation, and steroid use also play a role in diaphragmatic dysfunction in COPD patients in addition to pulmonary hyperinflation [2–4]. In COPD, loss of muscle mass has been described as the key determinant of mortality, independent of lung function, smoking, and body mass index (BMI) [5].

In severe COPD patients with FFM and muscle loss, the mass and thickness of the diaphragm may vary greatly. Autopsy examinations in these patients have shown reduced diaphragmatic thickness, volume, and surface compared to those without COPD [6]. Accordingly, evaluation of diaphragmatic function is getting more important in COPD patients. In these patients fluoroscopy is not a reliable approach for measuring diaphragmatic thickness; computerized tomography involves radiation exposure, and magnetic resonance imaging may yield better results, but it is more expensive. Therefore, ultrasonography appears to be a good option for measuring diaphragmatic dysfunction [4,7]. Although ultrasonographic measurement of diaphragmatic thickness is a simple, accurate, affordable, and repeatable method, sufficient information is not available at this time to establish whether it represents a significant indicator of severity of disease, symptoms, quality of life, and mortality.

To our knowledge, there are few reports available concerning the relationship between diaphragm mobility and COPD severity. There are also several methods for measuring the function of the diaphragm, and which sonographic method is most effective has not yet been defined. For this reason, contradictory results have been reported in the literature.

In this study, the diaphragmatic thicknesses of patients with moderate and severe COPD were measured using ultrasonography and compared with healthy controls in order to evaluate the relationship between the thickness of the diaphragm and the severity of parameters and clinical characteristics of the disease. 

## 2. Materials and methods

### 2.1. Study design and setting

This observational case-control study was carried out at a large tertiary referral academic institution after receiving institutional review board approval (No. 20171207-2). All patients gave verbal and written consent. The study included stable COPD patients admitted from December 2017 to February 2018. The diagnosis of COPD was based on an investigation of their medical history, a clinical examination, and respiratory function tests. COPD diagnoses of the patients were made based on the Global Initiative for Chronic Obstructive Lung Disease (GOLD) criteria. The patients were asked about smoking, exposure history, annual number of exacerbations, presence of concomitant conditions, and duration of the disease. The symptom scores were captured using the modified Medical Research Council Dyspnea Scale (mMRC). Patients older than 40 years who had a postbronchodilator forced expiratory volume in the first second/forced vital capacity (FEV1/FVC) ratio of <70% on pulmonary function testing (PFT) (Vmax Encore PFT System, USA), measured by the same trained operator according to ATS standards, were enrolled in the study. Serum glucose, urea, creatine, albumin, lipid profile, electrolytes, hemogram, B12, and folic acid levels were recorded. Echocardiographic (ECO) evaluation, ejection fractions (EF), and pulmonary artery pressure (PAP) levels were noted. Thirty-four patients with COPD and 34 healthy subjects who had been admitted to a university hospital were enrolled. Patients with acute exacerbation of COPD, malignancy, neuromuscular conditions, cerebrovascular disease, unilateral or bilateral pleural effusion, pneumothorax, atelectasis, pneumonia, interstitial lung disease, or a recent surgical operation and those who did not consent to participating in the study were excluded. Comorbidities, including cardiac insufficiency, hypertension, renal insufficiency, and diabetes mellitus, were asked about and recorded. 

### 2.2. Measurements

All sonography exams were performed when patients were in a supine position before and after deep inspiration. Both arms were positioned higher than the neck. The junction of diaphragmatic branches to the interior side of lateral chest walls were identified through the intercostal space by an axial view. The probe was rotated 90 degrees to see the diaphragmatic branches as parallel to the probe beams. The thickness of the right and left branches was measured before patients took a deep breath. Measured diaphragm thicknesses were recorded as TminR (right) and TminL (left). Patients were requested to take a deep breath and hold it for 10 to 20 s during the evaluation for both sides. The thicknesses of the branches were measured after holding the inflated chest still. The measurements were recorded as TmaxR (right) and TmaxL (left). A high-resolution linear probe (Voluson, General Electric Imaging, USA) with gray-scale imaging was used by one experienced radiologist. Three consecutive measurements were taken before and after deep inspiration and the average value of that series was calculated and recorded as a final result for both sides. All evaluations were made by the same radiologist who was blinded to the pulmonary function status of each patient.

The patients were divided into 4 groups, as Group A, Group B, Group C, and Group D, based on symptom score and number of exacerbations. Based on spirometry, patients with a FEV1 value of >80% were classified as having mild COPD, between 80% and 50% as having moderate COPD, between 49% and 30% as having severe COPD, and <29% as having very severe COPD.

### 2.3 Statistical analysis

SPSS 21 (IBM Corp., Armonk, NY, USA) was used for statistical analysis. Data are presented as mean ± SD or n (%), as appropriate. Correlations between continuous variables were tested using Spearman’s rho. Normality was tested using the Shapiro–Wilk Test. Student’s t-test was used for parametric variables and the Mann–Whitney U test was used for nonparametric variables for the differences between 2 groups in terms of Tmin–Tmax (diaphragm thickness). P < 0.05 was considered an indication of statistical significance.

## 3. Results

The 34 COPD patients, 29 males and 5 females, who were enrolled in the study had an average age of 71 ± 9 years and an average disease duration of 7.1 ± 5.4 years. There were 9 patients in GOLD Group C and 25 in Group D. Fourteen cases were categorized as moderate, 15 as severe, and 5 as very severe based on respiratory function. There were 8 patients with minor symptoms and 26 with advanced symptoms based on the symptom scores (mMRC). According to number of exacerbations, 27 patients had 2 or fewer, and 7 patients had more than 2. Patient demographics and clinical characteristics are shown in Table 1. 

**Table 1:** Demographic and clinical characteristics of patient and control groups.

	COPD group (n: 34)	Control group (n: 34)	P
Sex, M/F	29/5	20/14	0.001
Age (mean ± SD), years	71.0 ± 9.2	65 ± 7.1	0.012
Body mass index (mean ± SD), kg/m2	25.8 ± 4.9	27.5 ± 3.7	0.08
Smoking pack-years (mean ± SD)	39.2 ± 21	17.6 ± 17.6	0.001
Smoking (none/current/former)	4/1/29	14/9/11	
Number of exacerbations within the last year, mean ± SD [min/max]	1.94 ± 1.3 [1/6]		
FEV1/FVC, mean ± SD [min/max]	53 ± 10.2 [29/70]		
FEV1, mean ± SD [min/max] (% pred.)	45.1 ± 14.6 [20/73]		
mMRC score, mean ± SD	2 ± 0.7		
GOLD class A/B/C/D	-/-/9/25		
Spirometric class (moderate/severe/very severe), n	14/15/5		
pCO2, mean ± SD [min/max]	40.6 ± 11 [25.7/87.2]		
pO2, mean ± SD [min/max]	56.7 ± 9.3 [33.5/78.2]		
Saturation O2, mean ± SD [min/max]	89.8 ± 4.7 [78/96]		

FEV1: Forced expiratory volume in the first second; mMRC: modified Medical Research Council Dyspnea Scale; GOLD: Global Initiative for Chronic Obstructive Lung Disease.

Diaphragmatic thickness was measured separately on the left and the right side, both during tidal volume (Tmin) and deep inspiration (Tmax), and the measured results were correlated. There was no significant difference between the patient and control groups in diaphragmatic thickness. The results are shown in Table 2. 

**Table 2:** Comparison of groups in terms of diaphragm thickness.

Thickness mm	COPD group (n: 34)	Control group (n: 34)	P
TminR	2.74 ± 0.4	2.98 ± 0.8	0.134
TminL	2.77 ± 0.4	2.70 ± 0.7	0.647
TmaxR	4.40 ± 0.8	4.47 ± 0.8	0.721
TmaxL	4.41 ± 0.7	4.44 ± 0.7	0.905

TminR: Minimum thickness right; TminL: minimum thickness left; TmaxR: maximum thickness right; TmaxR: maximum thickness left.

There was no correlation between diaphragmatic thickness and COPD severity, respiratory function, frequency of exacerbations, smoking, or demographics. The results are shown in Table 3. 

**Table 3:** Correlations of diaphragm thickness with demographic and clinical data.

	TminR		TmaxR	
Characteristics	r	P	r	P
GOLD group	–0.09	0.6	0.06	0.7
Spirometric function	–0.33	0.06	–0.03	0.8
Frequency of exacerbations	–0.18	0.3	–0.04	0.8
Duration of COPD	–0.22	0.2	–0.16	0.3
FEV1	0.19	0.3	–0.18	0.3
mMRC score	–0.13	0.5	–0.01	0.9
pCO2	–0.02	0.9	0.05	0.8
pO2	–0.12	0.5	–0.01	0.9
Saturation O2	–0.14	0.4	–0.05	0.8
Age	–0.14	0.4	–0.28	0.1
Smoking status	0.26	0.1	0.24	0.2
BMI	0.08	0.7	0.24	0.2
EF	–0.08	0.7	0.10	0.6
PAP	–0.123	0.5	0.01	0.9

FEV1: Forced expiratory volume in the first second; mMRC: modified Medical Research Council; GOLD: Global Initiative for Chronic Obstructive Lung Disease; BMI: body mass index; EF: ejection fraction; PAP: pulmonary artery pressure.

When the patients were divided into 2 groups based on the number of annual exacerbations, respiratory severity, GOLD stage, symptom severity, and disease duration, we could not find a significant difference in terms of diaphragmatic thickness between the groups. The results are shown in Table 4. 

**Table 4:** The relationship between diaphragm thickness and COPD severity.

	n	Tmin R	P	Tmax R	P
Frequency of exacerbations					
<2	17	2.83 ± 0.5	0.188	4.60 ± 0.9	0.171
≥2	17	2.64 ± 0.4		4.21 ± 0.7	
GOLD group					
C	9	2.80 ± 0.6	0.779	4.32 ± 0.9	0.667
D	25	2.72 ± 0.3		4.44 ± 0.8	
mMRC					
<2	8	2.91 ± 0.6	0.328	4.49 ± 0.8	0.839
≥2	26	2.66 ± 0.4		4.38 ± 0.8	
FEV1					
<50%	20	2.66 ± 0.4	0.290	4.48 ± 0.8	0.410
≥50%	14	2.86 ± 0.5		4.30 ± 0.8	
Duration of COPD					
<10 years	22	2.79 ± 0.4	0.337	4.52 ± 0.8	0.295
≥10 years	12	2.65 ± 0.4		4.20 ± 0.9	
Comorbidities					
≤2	28	2.72 ± 0.4	0.702	4.38 ± 0.8	0.642
>2	6	2.80 ± 0.5		4.55 ± 0.7	

FEV1: Forced expiratory volume in first second; mMRC: modified Medical Research Council; GOLD: Global Initiative for Chronic Obstructive Lung Disease.

There was no signification correlation between the patients’ serum albumin, urea, creatine, magnesium, calcium, B12, folic acid, cholesterol, and triglyceride levels and diaphragmatic thickness. 

## 4. Discussion

Contrary to our expectations, there was no significant difference in terms of diaphragmatic thickness between the control group and the group of patients with moderate and severe COPD. Additionally, ultrasonographic measurement of diaphragmatic thickness, although an affordable, quick, and repeatable method, was insufficient in identifying those with high symptom scores and high risk of exacerbation. There was no relationship between diaphragmatic thickness and disease severity, disease duration, FEV1, symptom score, and frequency of exacerbations. Moreover, there was no correlation with concomitant conditions, blood gas values, and serum biochemistry parameters. 

Although it has been known for approximately 4 decades that the diaphragm is affected in COPD, the number of studies measuring diaphragmatic function is relatively low due to reasons such as the absence of standardization of the measurement methods [6,7]. Although patients’ diaphragmatic function is affected, studies have reported conflicting results, due to the complex functionality of the diaphragmatic muscle and nonstandardized measurement methods. For example, Baria et al. measured diaphragmatic thickness by ultrasonography in 50 COPD patients and 150 healthy controls and reported the absence of a significant difference in diaphragmatic thickness between the COPD patients and the controls. This study did not investigate the relationship of diaphragmatic thickness with clinical characteristics [8]. Eryüksel et al. did not identify a significant difference between diaphragmatic thickness fraction and disease severity, symptom severity, frequency of episodes, FEV1 value, and BMI [9]. Similarly, a study by Cimsit et al. could not establish a correlation between clinical parameters and diaphragmatic thickness [10]. However, the last 2 studies did not include a control group. In contrast, Smargiassi et al. reported that their thickness measurements by echocardiography were related to hyperinflation and lung volume. Additionally, the FFM, BMI, and BODE (BMI-Obstruction-Dyspnea-Exercise) index were correlated with diaphragmatic thickness. However, only 23 patients were evaluated [4].

Even though there were contradictory results with diaphragmatic thickness, studies involving measurement of the diaphragm on the craniocaudal plane during inspiration and expiration (excursion/lung silhouette) have reported more consistent results. Scheibe et al. reported that ultrasonographic measurement of lung silhouette movement was useful and reliable in demonstrating diaphragmatic dysfunction in patients with COPD. This study showed a strong correlation between diaphragmatic movement and FEV1 [3]. In a study comparing 25 COPD patients with 25 healthy controls, Davachi et al. detected statistically significant differences between the 2 groups in terms of diaphragmatic mobility. Also, in this study, diaphragmatic mobility was linked with airway obstruction [11]. Similarly, Paulin et al. observed reduced diaphragmatic mobility in patients with COPD compared to healthy controls. In this study, diaphragmatic mobility was related to hyperinflation. Those with lower diaphragmatic mobility were more dyspneic and had shorter 6-min walk distances [12]. In a study by Ünal et al., the travel difference between the diaphragmatic points was 26 mm for patients with COPD and 69 mm for healthy subjects using MR fluoroscopy. Also, excursion was correlated with FEV1 [13]. In a study of 37 COPD patients, Kang et al. reported a positive correlation for diaphragmatic mobility with FEV1 and FVC, and negative correlation with RV, TLC, and PaCO2 [14]. In a similar study, there was a reduction of the diaphragm movement distance compared to healthy subjects, and a correlation was described between diaphragm movement and airway obstruction (FEV1) [15].

Based on our findings and those of similar studies, we have reached the conclusion that diaphragmatic dysfunction in COPD is related to mobility restriction, rather than muscle thickness, which was the basis of our study hypothesis. Pulmonary hyperinflation affects excursion more prominently than thickness. Perhaps, contrary to our original proposition, diaphragmatic thickness is not adversely affected in COPD, due to overworking against an increased mechanical load. Chronic load may lead to adaptation, as with the skeletal muscle. Nevertheless, further investigation of the respiratory muscle function over time appears to be warranted. 

Our study had a number of limitations. First, we were unable to measure lung volumes. Data such as residual volume (RV) or total lung capacity (TLC) to prove hyperinflation were not available to the investigators. However, all of our patients were in Groups C and D, moderate and severe COPD patients, and most of them had clinical/radiological evidence of hyperinflation. Second, we were unable to perform bioelectrical impedance analysis to prove loss of muscle mass. In particular, evaluation of patients with notable cachexia and low FFM in the different groups could provide useful data, whereas we were able to use only BMI data and failed to identify a significant difference.

There has been growing interest in chest ultrasonography in recent years. Its area of use is expanding, particularly in diagnosis and follow-up of respiratory conditions including pneumothorax, acute and chronic interstitial diseases, pneumonia, and pleural effusion. Even though we have no significant results, our study has shown that the establishment of a proven area of use in COPD may benefit clinical practice. Further investigations are necessary for standardizing the technique and supporting selection of appropriate parameters for determining disease severity, risk of exacerbation, and mortality prediction.

## References

[ref0] (1990). Factors influencing ventilatory muscle recruitment in patients with chronic airflow obstruction. American Review of Respiratory Disease.

[ref1] (2005). Diaphragm dysfunction in chronic obstructive pulmonary disease. American Journal of Respiratory and Critical Care Medicine.

[ref2] (2015). Sonographic evaluation of diaphragmatic dysfunction in COPD patients. International Journal of Chronic Obstructive Pulmonary Disease.

[ref3] (2014). Ultrasonographic assessment of the diaphragm in chronic obstructive pulmonary disease patients: relationships with pulmonary function and the influence of body composition - a pilot study. Respiration.

[ref4] (2002). Midthigh muscle cross-sectional area is a better predictor of mortality than body mass index in patients with chronic obstructive Pulmonary disease. American Journal of Respiratory and Critical Care Medicine.

[ref5] (1973). Size of the diaphragm in chronic bronchitis. Thorax.

[ref6] (2016). A review of the ultrasound assessment of diaphragmatic function in clinical practice. Respiration.

[ref7] (2014). B-mode ultrasound assessment of diaphragm structure and function in patients with COPD. Chest.

[ref8] (2017). Diaphragmatic thickness fraction in subjects at high-risk for COPD exacerbations. Respiratory Care.

[ref9] (2016). Ultrasound assessment of diaphragm thickness in COPD. Marmara Medical Journal.

[ref10] (2014). The relationship between diaphragmatic movements in sonographic assessment and disease severity in patients with stable chronic obstructive pulmonary disease (COPD). Journal of Cardio-Thoracic Medicine.

[ref11] (2007). Influence of diaphragmatic mobility on exercise tolerance and dyspnea in patients with COPD. Respiratory Medicine.

[ref12] (2002). Evaluation of diaphragmatic movement with MR fluoroscopy in chronic obstructive pulmonary disease. Clinical Imaging.

[ref13] (2011). Influence of diaphragmatic mobility on hypercapnia in patients with chronic obstructive pulmonary disease. Journal of Korean Medical Science.

